# A Review of the Potential Interaction of Selenium and Iodine on Placental and Child Health

**DOI:** 10.3390/nu12092678

**Published:** 2020-09-02

**Authors:** Nahal Habibi, Jessica A. Grieger, Tina Bianco-Miotto

**Affiliations:** 1 School of Agriculture, Food and Wine, Waite Research Institute, and Robinson Research Institute, University of Adelaide, Adelaide 5005, Australia; nahal.habibi@adelaide.edu.au; 2 Adelaide Medical School, and Robinson Research Institute, University of Adelaide, Adelaide 5005, Australia

**Keywords:** iodine, micronutrients, selenium, pregnancy, placenta, oxidative stress

## Abstract

A healthy pregnancy is important for the growth and development of a baby. An adverse pregnancy outcome is associated with increased chronic disease risk for the mother and offspring. An optimal diet both before and during pregnancy is essential to support the health of the mother and offspring. A key mediator of the effect of maternal nutrition factors on pregnancy outcomes is the placenta. Complicated pregnancies are characterized by increased oxidative stress in the placenta. Selenium and iodine are micronutrients that are involved in oxidative stress in placental cells. To date, there has been no comprehensive review investigating the potential synergistic effect of iodine and selenium in the placenta and how maternal deficiencies may be associated with increased oxidative stress and hence adverse pregnancy outcomes. We undertook a hypothesis-generating review on selenium and iodine, to look at how they may relate to pregnancy complications through oxidative stress. We propose how they may work together to impact pregnancy and placental health and explore how deficiencies in these micronutrients during pregnancy may impact the future health of offspring.

## 1. Introduction

Pregnancy is characterized by a pro-inflammatory, hyperlipidemic and hyperinsulinemic state [1]. A normal healthy pregnancy is important for the growth and development of the baby and for the lifelong health of the mother and offspring [2]. To provide a suitable condition for the developing fetus, the maternal body undergoes major biochemical, physiological and anatomic adaptations [3], which typically revert back to the condition they were in prior to pregnancy [4].

Human pregnancy is also associated with an increase in oxidative stress markers, due to increased placental mitochondrial activity and the production of reactive oxygen species, which have pronounced effects on placental function [5]. The placenta is the interface between the mother and baby. It mediates nutrient and waste exchange to support a healthy pregnancy. Factors such as poor maternal diet or obesity impact placental health and function, such as proliferation, invasion and apoptosis of trophoblast cells [6,7]. This can have a profound effect on fetal growth and development, and pregnancy success [6,7]. As such, a balance of antioxidant and oxidant status is key to a healthy placenta and hence a successful pregnancy outcome for mother and baby.

There is a clear role for an optimal diet both before and during pregnancy to support the health of the mother and offspring [8,9,10,11,12]. Higher dietary scores for a high-protein/fruit pattern before pregnancy, was associated with decreased likelihood of preterm birth (adjusted odds ratio; 95% confidence interval, OR: 0.31; 95% CI: 0.13, 0.72), whereas the reverse direction was apparent for the high-fat/sugar/takeaway pattern (adjusted OR: 1.54; 95% CI: 1.10, 2.15) [13]. Increasing dietary quality, calculated by the Alternative Healthy Eating Index 10 during pregnancy, was associated with a decrease in the likelihood of delivering a small-for-gestational-age (SGA) baby [14]. Supplementation with vitamins and minerals may also have a positive effect on infant birthweight [15]. Some micronutrients, such as selenium and iodine, are essential micronutrients because the body cannot produce them and the mother needs to obtain them from her diet or from supplemental intake. Deficiencies in iodine can lead to thyroid hormone deficiency, and therefore improper neurodevelopment and mental retardation in the fetus [16]. Selenium deficiency is associated with pregnancy-induced hypertensive disorders [17], miscarriage [18], preterm birth [19], and gestational diabetes [20,21].

Importantly, the key mediator of the effect of maternal nutrition factors on pregnancy outcomes is the placenta. However, such pregnancy outcomes do not just end after pregnancy, but can have lifelong consequences for a mother and her offspring. This is depicted by the developmental origins of health and disease (DOHaD) hypothesis, which emphasizes the importance of fetal life exposure to maternal factors, such as diet, in the development of chronic diseases later in life [22]. Yet, while maternal nutrition and the placenta are important factors regarding pregnancy success, there is a paucity of information examining multiple nutrients in combination, and the potential synergistic effect they may have in pregnancy.

**Figure 1:**
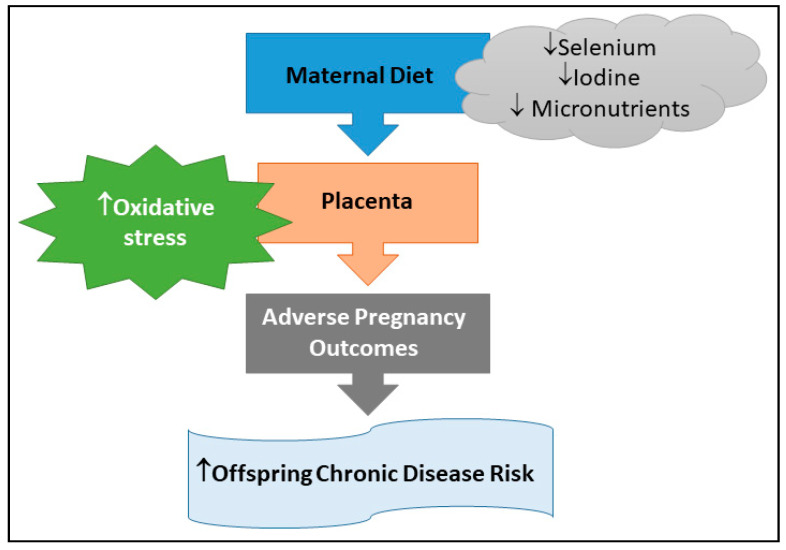
Deficiencies (↓) in selenium and iodine result in placental oxidative stress which may contribute to adverse pregnancy outcomes and hence increased (↑) risk of chronic disease in offspring.

Adverse pregnancy outcomes are associated with increased oxidative stress in the placenta [23] and since selenium and iodine are involved in oxidative stress in placental cells [24], it is important to investigate the role of both micronutrients. To the best of our knowledge, no study has reviewed current evidence regarding the potential synergistic effect of iodine and selenium in the placenta. Our knowledge from other organs, such as the thyroid, highlights the importance of these two micronutrients and their combined impact on oxidative stress [25]. We identified studies that investigated the association between maternal selenium and iodine status with pregnancy complications using the following keywords: “maternal blood selenium concentration AND pregnancy complication” and “maternal urinary iodine concentration AND pregnancy complication” in PubMed. The last search was performed in July 2020. In addition, any relevant studies identified from the reference list of selected studies were also included. The exclusion criteria were: (1) review papers, (2) unavailable full text, (3) non-English language, (4) animal studies, and (5) taking any medication during pregnancy. Pregnancy outcomes were defined as healthy if there was no complication or complicated, if there was one of the following pregnancy complications: preeclampsia (PE), pregnancy-induced hypertension, miscarriage, gestational diabetes mellitus (GDM), preterm birth (PTB), intrauterine growth restriction (IUGR), or premature rupture of membrane (PROM). In this review we outline how selenium and iodine may relate to pregnancy complications through oxidative stress, and we propose how they may work together to impact pregnancy and placental health (Figure 1). We also explore how deficiencies in these two micronutrients during pregnancy may impact on the future health of the offspring, which has not been previously reviewed as a combination.

## 2. Oxidative Stress and the Importance of Antioxidants

Oxidative stress occurs when free radicals are generated to a level higher than the physiological level and the antioxidant system cannot neutralize them [26,27]. Free radicals, namely reactive oxygen species (ROS), are unstable molecules that have unpaired electrons and can donate or accept an electron to be stabilized [28]. As a result, the new molecule is unstable and formation of free radicals continues. This chain reaction can cause oxidative damage to nucleic acids, lipids, carbohydrates and proteins, which results in the decaying of tissues and the disruption of homeostasis [27]. The production of ROS is a continuous phenomenon in cells and is essential for cell-signaling, however, excessive ROS levels are detrimental to cells [27,29].

To stop the accumulation and damage of ROS, free radicals need to be neutralized by pairing unpaired electrons. This is how the antioxidant system can protect the cell against oxidative damage [30]. Antioxidants typically work in three ways, including the prevention of ROS formation, the interception of a ROS chain reaction and the repairing of damaged molecules [31,32]. The antioxidant defense system is made up of enzymes and non-enzyme components. Glutathione peroxidase (GPx), catalase, superoxide dismutase and thioredoxin reductase (TRx) are some of the enzymes [33] but they need cofactors such as micronutrients to work properly. For example, superoxide dismutase requires manganese or copper and zinc, while GPx and TRx need selenium to function properly. Therefore, micronutrient deficiencies can reduce the ability of the antioxidant system to protect cells against free radicals [34]. Vitamin C, vitamin E and beta-carotene are other micronutrients involved in the antioxidant defense system [35,36].

### Oxidative Stress and Pregnancy Complications

Pregnancy complications are adverse outcomes of pregnancy such as preeclampsia (PE), gestational diabetes mellitus (GDM), intrauterine growth restriction (IUGR), preterm birth (PTB), and premature rupture of membrane (PROM). They can increase morbidity and mortality rates for both the mother and her offspring [37,38,39,40].

Preeclampsia is a pregnancy-specific, multisystem disorder, presenting as hypertension with new onset of one or more of proteinuria or renal, liver, neurological or hematological complications, or uteroplacental dysfunction after 20 weeks’ gestation [41]. Patients with a history of PE have around a 2-fold increased risk for vascular diseases such as hypertension and ischemic heart disease [42], stroke [42] and chronic kidney disease [43], at 10–15 years post-partum. Infants of mothers who had PE are also at an increased risk of small-for-gestational-age [44] and perinatal mortality [45]. Children exposed to preeclamptic pregnancies have increased hospitalizations due to infectious and parasitic, nutritional and metabolic diseases of the respiratory system, blood, and blood-forming organs at ages 1–13, 16, 18–21 and 24 years compared to unexposed children [46]. Moreover, women who experience PE in their first pregnancy have a higher risk of myocardial infarction and cardiovascular death compared to non-PE mothers [47]. Currently, delivery of the fetus and placenta is the only treatment [41,42,45,48,49].

The pathogenesis of PE involves improper placental development as a result of dysfunctional proliferation, migration, and invasion of placenta-derived extravillous cytotrophoblast cells into the uterine vasculature, along with maternal endothelial and vascular dysfunction [48]. This leads to placental hypoxia and subsequent reperfusion, resulting in oxidative stress and inflammation [50,51].

Similar to PE, oxidative stress also contributes to PTB, IUGR and PROM. IUGR refers to a fetus that is smaller than expected for their gestational age. One of the most common causes of IUGR is uteroplacental insufficiency in which the placenta is unable to provide the developing fetus with sufficient nutrients and oxygen. The growth-restricted offspring have a 2–6 fold increased risk of developing chronic diseases such as type 2 diabetes mellitus, coronary heart disease and chronic kidney disease later in life [52].

The same placental dysfunction seen in PE is also seen in IUGR with deficient spiral artery remodeling resulting in malperfusion [53]. The malperfusion results in oxidative stress within the placenta and overwhelms the antioxidant system. In addition, the enzymes which act as antioxidants require micronutrients to work and maternal diets deficient in these micronutrients impact on the placenta’s ability to combat oxidative stress [53].

## 3. Micronutrients and Pregnancy

To compensate for the higher demand of growing maternal tissues and fetus and also for hemodilution that occurs in pregnancy, a higher dietary intake of many micronutrients and trace elements are recommended [4,54]. In developed countries, despite the availability of suitable nutritional foods, many pregnant women have an imbalanced diet that puts them at risk of an inadequate intake of micronutrients like folate, vitamin D, vitamin B12, iron and iodine [55,56]. There is considerable evidence that shows that deficiency of micronutrients such as iodine, selenium, zinc, vitamin E, folate and iron, adversely impacts maternal and fetal health, and pregnancy outcome [19,57,58,59,60,61,62,63,64,65,66,67,68,69]. One of the reasons for this association may be the bio-functionality of vitamins and minerals in pregnancy, besides their classical roles in health and disease in the general population [56]. Although maternal micronutrient intake through the entire gestational period can affect fetal growth and development, the peri-implantation stage and placental development are critical windows for programming a healthy birth and future life [70,71].

### 3.1. Selenium

Selenium is an essential trace element that plays a pivotal role in the antioxidant defense system, cell cycle, and immune function, because of its contribution to selenoproteins [72]. Selenoproteins such as glutathione peroxidases (I, II, III, IV, and VI); thioredoxin reductases (I, II, and III); and selenoprotein H, P, and W, have antioxidant properties and are involved in managing ROS production. Selonoproteins also possess a vast range of functions such as protein folding, signaling, lipid biosynthesis, cell cycle, and calcium regulation [73,74,75,76,77,78,79].

Selenium is found in a broad range of foods including brazil nuts, wholegrain foods and cereals, fish, beef, eggs, and some fortified ready-to-eat breakfast cereals. The recommended dietary intake of selenium has been calculated by the amount of selenium needed to maximize the synthesis of glutathione peroxidase (GPx) [80]. During pregnancy, it is 60 micrograms per day in European countries [81] and 65 micrograms per day in Australia and New Zealand [82]. The requirement for selenium is higher during pregnancy, up to 4 micrograms per day, due to fetal requirements [83].

Globally, up to one in seven people have a low selenium dietary intake [84]. Selenium levels can be influenced by multiple factors including drinking water, soil, plant and animal tissue content of selenium [84], as well as different intakes in different regions. For example, the average intake of selenium in Eastern Europe is lower than that of Western Europe [85]. In addition, in New Zealand [85] and most areas of China [86], selenium deficiency is evident. In the Middle East, selenium intake is dependent on socio-economic status [85].

While studies across different countries report various levels of selenium during pregnancy (Table 1), using the data reviewed by Mariath et al. (2011) [87], Perkins and Vanderlelie (2016) proposed that selenium levels below 45 micrograms per litre can be dangerous and can be associated with adverse birth outcomes. They further concluded that selenium levels above 95 micrograms per litre can be considered as seleno-sufficient because the majority of selenoproteins can be maximally expressed at this level [57]. In addition to the measurement of maternal selenium levels, markers of functional effects of selenium may provide additional information. For instance, assessment of selenoproteins such as GPX3 and SEPP1 are suitable to investigate the functional effects of selenium such as antioxidant activity, nutritional selenium deficiency, and evaluating responses of deficient individuals to selenium supplementation [88]. However, more studies would be needed to determine whether these selenoproteins provide additional information for interpreting clinical outcomes in addition to, or instead of, maternal selenium levels. The time point of selenium assessment can also affect the interpretation of the data, since pregnant women that were selenium sufficient in the first trimester have been shown to become selenium deficient later in pregnancy [89].

### 3.2. Selenium and Pregnancy Complications

Selenium is necessary for human reproduction [100]. We have recently shown that in 1060 Australian pregnant women, those who had lower selenium concentrations (<0.95 micromol per litre) took a longer time to conceive (1.19 (1.01–1.40)), an equivalent of around 0.6 months, and they were at greater risk of infertility (1.46 (1.06–2.03)) [101]. Deficiency in selenium, measured by lower concentrations either in plasma, toenail, red blood cells or hair, has also been associated with a higher risk of pregnancy complications such as pregnancy-induced hypertension, PTB, impaired glucose tolerance and GDM [19,20,21,67,95,96,97,99,102,103] (Table 2). Comparatively, selenium supplementation was associated with a lower incidence of PE and PROM [104,105,106]. Importantly, inflammatory indicators such as high sensitivity C-reactive protein were higher in selenium-deficient pregnant women and antioxidant defense indicators namely GPx, catalase, thiobarbituric acid reactive substances (TBARS), superoxide dismutase, and glutathione S-transferase were altered [20,95,99,107,108] (Table 2). This supports that selenium deficiency may contribute to higher oxidative stress levels, inflammation, and subsequent pregnancy complications (Table 2).

### 3.3. Iodine

Iodine is a vital trace element required for thyroid hormone synthesis and plays a fundamental role in fetal brain development [109,110]. Maternal iodine deficiency during pregnancy is associated with insufficient neurodevelopment, and defective intellectual skills such as poorer school performance and language delay as well as attention-deficit/hyperactivity disorder in offspring later in life [111,112]. While iodine deficiency is described as the most common cause of a child’s central nervous system maldevelopment [113], mandatory iodine fortification programs such as salt iodization have helped address this common health issue among women of child-bearing age globally [114,115,116,117]. In Australia, although this preventive program seems to be successful among the general population, 16–44 year old pregnant women’s median urinary iodine concentrations (MUIC: 116 micrograms per litre) still indicate insufficient iodine intake [118]. In New Zealand, MUIC of 68 micrograms per litre indicates a mild iodine deficiency in 18–44 year old women post-mandatory iodine fortification [118]. A similar situation has been reported in other countries with iodine fortification such as Austria, Croatia, Egypt, and Iran, all reporting that pregnant women’s iodine status was insufficient [119]. Pregnancy iodine status should be at an optimal level to avoid the potentially harmful consequences of iodine deficiency [120].

Iodine intake during pregnancy should cover the needs of both the mother and her developing fetus. Thus, it is recommended that dietary intake increases from 150 micrograms perday for a non-pregnant woman to 220 micrograms per day during pregnancy [82]. The World Health Organization recommends a daily iodine supplement of 250 micrograms per day during pregnancy, or an annual dose of iodized oil supplement of 400 milligrams per year [121]. While maternal dietary intake of iodine can impact placental iodine content, and therefore control the effect of iodine on fetal thyroid gland activity [122], placental iodine accumulation plays a significant role in iodine availability to the fetus [123].

In thyroid hormone production, I^_^ oxidation occurs to form iodine (I_2_). This reaction uses H_2_O_2_ and thyroid peroxidase and inhibits H_2_O_2_ accumulation or its decomposition to a hydroxyl radical [25,124]. A normal level of thyroid hormone exerts a negative feedback on thyroid-stimulating hormone (TSH). Iodine deficiency is associated with several health issues including a reduction in thyroid hormone production [125], which results in the absence of the negative feedback on TSH. Therefore, a cascade of signals and reactions, including increasing TSH secretion, occurs [126]. TSH stimulates H_2_O_2_ generation for I^−^ oxidation but in severe iodine deficiency this process does not occur, resulting in thyroid hormone insufficiency [125,126]. Therefore, TSH continues to increase H_2_O_2_ generation, which can be higher than the antioxidant capacity of GPx; H_2_O_2_ will accumulate and more ROS is produced, resulting in oxidative stress and apoptosis [126].

Iodide is the ionic state of iodine, occurring when iodine forms a salt with another element, such as potassium. Iodide may have an ancestral antioxidant function in various iodide-concentrating cells not only similar to thyroid cells where iodine consumes H_2_O_2_, but also because iodide can reduce the lipid peroxidation rate by reacting with double bonds of the cell membrane polyunsaturated fatty acids and make iodolipids that will be less reactive to ROS [127].

### 3.4. Iodine and Pregnancy Complications

There is limited but supporting evidence that iodine contributes to the antioxidant system. In a small sample of 74 women, there was decreased total antioxidant status and superoxide dismutase activity in women with mild iodine deficiency in the 2nd and 3rd trimesters of pregnancy compared to pregnant women with optimal iodine levels [138]. Iodine sufficient pregnant women, as indicated by more than 150 micrograms per litre urinary iodine concentration, had higher superoxide dismutase enzyme activity compared to iodine-deficient pregnant women [66]. In vitro studies support this and have shown that iodine supplementation reduces ROS production in a dose-dependent manner [139]. While to date there is no study about the potential antioxidant effect of iodine in the placenta, studies have shown a role for iodine in the antioxidant system of other organs such as thyroid, breast, stomach and eye [25,140,141,142,143]. We have recently shown in a placental cell line that treatment with iodine resulted in a lower lipid peroxidation compared to control upon induction of oxidative stress [24], further supporting a potential role of iodine as an antioxidant in the placenta.

Importantly, several studies have shown that iodine-deficient pregnant women are at an increased risk of pregnancy complications such as maternal high blood pressure, PE, IUGR and PTB [64,65,66,138,144,145] (Table 3). Iodine assessment methods can significantly impact the iodine measurement [146]. Iodine intake can be assessed indirectly by thyroid hormone level, urine spot or 24 h urinary samples [146]. Although spot urinary samples in population studies with more than 500 people is a more feasible approach and provides a good estimate of iodine status, it is not a valid measure for individual iodine status; 24 h urine analysis is a more appropriate assessment among individuals or in studies with smaller sample sizes [146]. Adjusting for urinary creatinine to avoid the influence of fluid intake also improves the reliability of the measurement of iodine status [146,147]. However, regardless of the assays used to measure iodine, it is clear that iodine deficiency is associated with pregnancy complications (Table 3). Because complications in pregnancy are exacerbated by increased oxidative stress, the role of iodine as an antioxidant may positively impact pregnant complications.

### 3.5. Potential Synergistic Effects of Selenium and Iodine

To date, most studies have investigated the impact of micronutrients on oxidative stress in the placenta, separately. Unfortunately, this is unlikely to provide more information on potential interactions or synergistic effects than if they were assessed in combination. In particular, selenium and iodine are essential micronutrients that may affect oxidative stress synergistically. Deiodinases are selenocysteine-containing enzymes that can regulate thyroid hormone bioavailability by removing iodide from different positions on the tyrosine ring. There are various deiodinases in different tissues (deiodinase I, II, III), however type III is dominant in the placenta. It inactivates the T3 (triiodothyronine) and T4 (thyroxine) hormones by removing an inorganic iodine from their inner ring and converting them to T2 (diiodothyronine) and reverse T3 (rT3), respectively [148,149]. Deiodinase type II can produce T3 from T4, and increase the bioavailability of T3 [150]. Thus, the activity of both deiodinase II and deiodinase III leads to the release of iodine in the placenta [151].

Selenoprotein expression in endocrine tissues is precisely controlled to be maintained, even in Se deficiency, and deiodinases seem to be higher than GPxs hierarchically in some tissues [152]. It is also unclear whether there are similar protective mechanisms for deiodinase in the placenta because of the lack of systematic investigation of the placental deiodinases and other selenoproteins. Only one study has reported that placental deiodinase III mRNA expression and its enzyme activity were correlated in PE but not in normotensive pregnant women [135]. In this study, maternal selenium levels were significantly lower in PE compared to normotensive women. This suggests that in normotensive pregnant women where selenium level is optimal, translation of deiodinase III is conserved, while in PE women with low selenium levels, deiodinase III enzyme regulation is altered; therefore iodine metabolism may be affected [135]. However, further studies are required and in larger sample sizes. This will help identify whether and how fetal adaptations occur, in order to maximize iodide uptake.

## 4. Impact of Maternal Selenium and Iodine, via Oxidative Stress, on Child Health

Maternal deficiencies in selenium or iodine may result in oxidative stress in the placenta, which may impact on future offspring health through developmental origins of health and disease [158,159,160,161]. Numerous studies support that an adverse in utero environment contributes to future chronic disease risk in adult offspring (reviewed in [162]) and that this may be mediated by the placenta [159,163].

It is known that during a healthy pregnancy there is a large amount of oxidative stress, especially since the placenta initially develops in a hypoxic environment with maternal blood flow established at approximately 10 weeks of gestation [164]. During this time, reactive oxygen species are produced and the antioxidant system combats this, however, if there are deficiencies in micronutrients involved in this antioxidant system, then it is likely that oxidative stress will occur which will lead to damaged placenta and potentially pregnancy complications. These pregnancy complications are associated with future chronic disease risk in the offspring. Several animal studies have shown that the use of maternal antioxidant supplementation can prevent placental oxidative stress and the associated programming of cardiovascular disease risk to the offspring (reviewed in [165]). Human randomized controlled trials with antioxidants have not shown improvements in pregnancy complications and some have in fact been associated with an increased risk (reviewed in [166]). However, what needs to be considered is the micronutrient status of the mother as deficiencies in elements such as selenium and iodine, which are required for antioxidant enzymes, may diminish the effectiveness of simply adding antioxidant supplements to the maternal diet.

## 5. Conclusions

**Figure 2:**
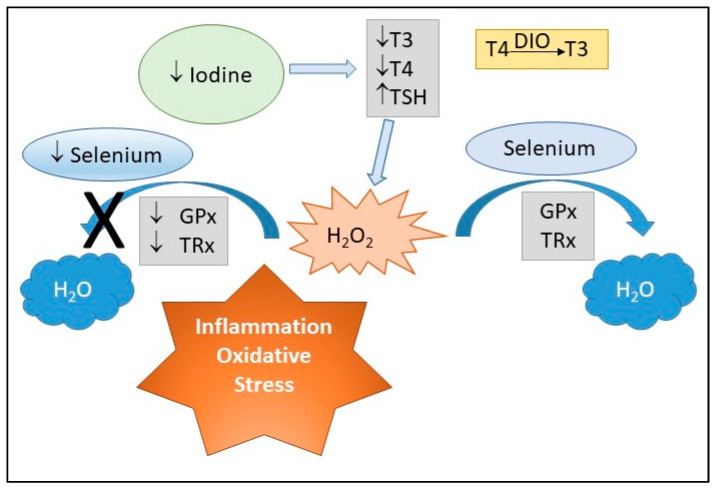
Deficiencies (↓) in iodine result in decreased (↓) T3 (triiodothyronine) and T4 (thyroxine) levels but increased (↑) TSH (thyroid-stimulating hormone) which results in more H_2_O_2_ (hydrogen peroxide). Antioxidants such as GPx (glutathione peroxidase) and TRx (thioredoxin reductase) are selenoproteins and in the presence of adequate selenium can convert H_2_O_2_ to H_2_O (water). However, if there is a selenium deficiency (↓), H_2_O_2_ accumulates and can result in increased oxidative stress and inflammation. Deiodinases (DIO) are selenoproteins and deiodinase II converts T4 to T3.

This is the first comprehensive review examining the potential synergistic effects of selenium and iodine (Figure 2). In addition, we have discussed whether the association between maternal selenium and iodine status as measured in biological specimens is associated with pregnancy complications due to their roles in oxidative stress. In future, studies that assess maternal dietary intake of selenium and iodine should also be examined. Iodothyronine deiodinases are selenoenzymes involved in thyroid hormone metabolism. The incorporation of selenium into deiodinases causes it to play an essential role in the metabolism of thyroid hormones and in the release of iodide. In addition, iodine deficiencies result in greater production of H_2_O_2_, which requires the selenoenzyme GPx to remove the excess H_2_O_2_. Thus, selenium and iodine may have some combined effects that should be investigated. Maternal diet is essential for the health of the placenta and baby, and deficiencies in micronutrients impact placental health potentially via oxidative stress pathways. This in turn not only increases the risk of an adverse pregnancy outcome, but is associated with poor future health outcomes for mother and offspring. Supplementation with antioxidants is not necessarily the key if the underlying selenium and iodine levels are low, as antioxidants require these and other micronutrients for optimal activity. Therefore, to address adverse pregnancy outcomes and the impact they have on future offspring health, a better understanding of the role of each micronutrient, alone and in combination, in placental development and hence pregnancy success is needed.

## Figures and Tables

**Table 1:** Selenium levels in women of reproductive age.

References (Ref.)	Country Year	Sample Size (Women)	Time of Sampling	Sample Type	Selenium Concentration (µg/L)
[90]	Indonesia 2019	25 pregnant	Delivery	Serum	76.42 ± 16.30
[91]	Turkey 2019	30 pregnant	Delivery	Blood, urine, amniotic fluid	Median (min-max):
					Maternal blood: 78.98 (72.36–84.14)
					Maternal urine: 23.44 (19.66–26.69)
					Amniotic fluid: 26.00 (22.56–29.88)
[92]	Australia 2018	558 pregnant	15 ± 1 weeks’ gestation	Plasma	Mean ± SD: 71.93 ± 11.05
[93]	Sudan 2014	31 pregnant	Not reported	Serum	Median (25–75th quartile): 204 (68–541)
[94]	Iran 2011	40 pregnant	34–39 weeks’ gestation	Blood	Mean ± SD: 58.51 ± 11.85
[95]	UK 2008		Delivery	Blood	Mean ± SD:
		27 pregnant			Pregnant: 58.4 ± 14.9
		22 non-pregnant			Non-pregnant: 69.8 ± 11.7
[20]	Hungary 2008		24–28 weeks’ gestation	Serum	Mean ± SD:
		20 pregnant			Pregnant: 40.5 ± 8.03
		24 non-pregnant			Non-pregnant: 77.4 ± 14.82
[96]	Turkey 2005		28–39 weeks’ gestation	Serum	Mean ± SD:
		28 pregnant			Pregnant: 87.50 ± 10.96
		25 non-pregnant			Non-pregnant: 109.0 ± 6.34
[97]	Kuwait 2004	15 pregnant	Delivery	Blood	Mean ± SEM:
					Maternal vein: 102.3 ± 3.1
					Umbilical artery: 85.4 ± 4.2
					Umbilical vein: 82.6 ± 4.1
[98]	USA 2004	22 pregnant	12 and 34 weeks’ gestation	Plasma	Mean ± SD:
					12 weeks’ gestation: 126.0 ± 15.0
					34 weeks’ gestation: 111.0 ± 12.0
[99]	Poland 2001		Before or within 12 hours after uterine curettage	Whole blood and plasma	Mean ± SD: Whole blood:
		36 pregnant			Pregnant: 74.1 ± 11.6
		28 non-pregnant			Non-pregnant: 90.5 ± 11.2
					Plasma:
		36 pregnant			Pregnant: 54.6 ± 11.1
		28 non-pregnant			Non-pregnant: 66.1 ± 13.1

SD, Standard deviation; SEM, standard error of the mean; µg/L, micrograms per litre.

**Table 2:** Association between maternal selenium levels or selenoproteins and pregnancy complications.

Ref.	Country Year	Sample Size (Pregnant Women)	Time of Sampling	Sample Type and Assays	Outcomes
[128]	Norway 2020	2638 (2558 term, 80 PTB)	17–18 weeks	Blood Se	No association between blood Se and PTB risk with adjustment for iodine intake (population had moderate iodine deficiency)
[90]	Indonesia 2019	51 (25 term, 26 PTB)	Delivery	Serum, placental and cord blood Se	Lower Se in the placenta and cord blood from PTB compared to term deliveries (*p* < 0.05)
[91]	Turkey 2019	50 (30 term, 20 PTB)	Delivery	Blood, urine and amniotic fluid Se	Lower blood, urine and amniotic fluid Se in PTB compared to term (*p* < 0.05)
[92]	Australia 2018	1065 (480 healthy, 585 complicated)	15 ± 1 weeks	Plasma Se, C-reactive protein	No difference between complicated and healthy pregnancies. No association between Se and C-reactive protein.
[129]	Iran 2017	60 at risk for IUGR (30 Se-supplementation, 30 placebo); RCT	Week 17 and end of intervention (week 27)	Plasma total antioxidant capacity, glutathione Serum C-reactive protein	Higher total antioxidant capacity and glutathione and lower C-reactive protein after 100 μg/d Se supplementation
[130]	South Africa 2017	66 (23 healthy, 43 PE)	Delivery	Serum and hair Se	Lower Se concentration in PE, no difference in hair Se between healthy and PE
[131]	Bangladesh 2015	74 PE (52 mild, 22 severe 118 normotensive)	≥20 weeks	Serum Se	Lower Se concentration in mild and severe PE, lower Se in severe PE compared to mild PE
[132]	Iran 2015	65 with GDM (32 Se-supplementation 33 placebo)	Week 24 and end of intervention (week 28)	Plasma MDA and glutathione	Higher glutathione and lower MDA after 200 μg/d Se- supplementation
[133]	Australia 2015	716 (472 healthy, 244 PE)	15 ± 1 weeks	Plasma Se	No difference in plasma Se between healthy and PE
[104]	UK 2015	230 primiparous 60 μg/d selenium yeast or placebo	(1) 12 and 35 weeks	(1) Whole-blood Se	After Se supplementation, low toenail Se was associated with decreased OR for PE/PIH (OR 0.30, 95% CI 0.09, 1.00, *p* = 0.049)
			(2) 16 weeks	(2) Toenail Se	
[93]	Sudan 2014	62 (31 healthy,31 GDM)	Not reported	Serum Se	No difference in Se level between healthy and GDM pregnancies
[106]	UK 2014	229 primiparous (115 Se-treated, 114 placebo) 60 μg/d selenium yeast	(1) 12 and 35 weeks	(1) Whole-blood Se	Higher Se and selenoprotein P in Se-treated group at 35 weeks. Reduced PE/PIH odds in all Se-treated participants (OR 0.350, 95% CI 0.126, 0.974; *p* = 0.044)
			(2) 35 weeks	(2) selenoprotein P	
[134]	Iran 2013	76 (38 healthy, 38 PE)	24 weeks–2 days after delivery	Plasma Se	Lower Se in women with PE compared to healthy pregnancies
[135]	UK 2013	50 (27 healthy, 23 PE)	Delivery	Serum Se, placental deiodinase mRNA and enzyme activity	Lower Se in women with PE, correlation between placental deiodinase III mRNA expression and its enzyme activity only in PE
[136]	Indonesia 2013	71 (46 healthy, 25 miscarriage)	8–20 weeks	Serum Se, GPx	Lower Se in women with miscarriage, no difference in GPx activity
[94]	Iran 2011	80 (40 healthy, 40 PE)	34–39 weeks	Plasma Se	Lower Se in women with PE
[19]	Netherlands 2011	1129 (60 PTB, 21 PROM, 13 PE)	12 weeks	Serum Se	Higher risk for PTB with lower Se (OR 2.18, 95% CI 1.25–3.77)
[105]	Iran 2010	166 primigravid (83 Se supplement, 83 placebo) 60 μg/d selenium yeast	1st trimester and delivery	Serum Se	Increased Se (*p* < 0.01) and lower incidence of PROM (*p* < 0.001) in 100 μg/d of selenium yeast-supplemented group
[95]	UK 2008	74 (27 healthy, 25 PE, 22 non-pregnant age-matched)	Delivery	Serum, plasma, umbilical venous Se. Plasma and umbilical venous TBARS. Plasma and placental GPx.	Decreasing trend of plasma Se from non-pregnant to normal pregnant and PE; Lower serum Se and plasma GPx in PE compared to healthy pregnancies; higher level of maternal and umbilical venous TBARS in PE group; Lower placental GPx in PE
[20]	Hungary 2008	61 (20 healthy, 17 GDM, 24 healthy non-pregnant)	24–28 weeks	Serum Se, high sensitivity C-reactive protein	Negative correlation between serum Se and high-sensitive C-reactive protein
[21]	Turkey 2008	180 (101 healthy, 30 GDM, 49 glucose intolerance)	24–28 weeks	Serum Se	Lower Se in GDM and glucose intolerance compared to healthy pregnancies
[137]	Kuwait 2007	20 obese (10 GDM, 10 control)	Delivery	Blood Se, GPx, SOD and total antioxidant enzyme activity	Higher SOD activity in maternal vein, umbilical artery and umbilical vein blood of control obese women
[96]	Turkey 2005	85 (32 PE, 28 healthy pregnant, 25 non-pregnant)	28–39 weeks	Serum and placenta MDA, SOD and catalase in erythrocytes. Placental GSH and GPx. Serum Se	Lower serum Se, erythrocyte SOD activity, and placental GPx and higher erythrocyte catalase activity in PE; negative correlation between placental MDA level and serum Se in PE
[67]	Italy 2005	504 (210 gestational hyperglycemic, 294 normoglycemic)	24–28 weeks	Serum Se Dietary intake of Se	Lower dietary intake of Se in gestational hyperglycemic women; lower serum Se in women with impaired glucose tolerance; negative association between Se (OR 0.92, 95% CI = 0.87 to 0.95, *p* < 0.0001) and gestational hyperglycemia
[97]	Kuwait 2004	30 (15 healthy, 15 GDM)	Delivery	Blood Se from maternal vein, umbilical artery and umbilical vein	Lower maternal vein Se in GDM compared to healthy pregnancies
[108]	Turkey2003	36 (16 healthy, 9 PIH and PE, 3 IDDP; 3 GDM, 3 OP, 2 PAP)	3rd trimester and immediately after delivery	GST, GPx and catalase activity and TBARS in maternal erythrocyte, plasma, and umbilical cord blood	Higher erythrocyte GPx activity and increased plasma TBARS in PIH and IDDP; higher cord blood GST activity (2–3 fold) in PE and PIH and IDDP compared to maternal activity before delivery; lower cord blood GPx activity compared to before delivery in PE and PIH; lower cord blood GPx (in PE and PIH, IDDP) and catalase activity (in PE and PIH) compared to maternal values; higher plasma TBARS in PE and PIH and IDDP in the antenatal period; lower cord blood erythrocytic TBARS in PE and PIH compared to maternal value
[102]	UK 2003	106 (53 healthy, 53 PE)	Not reported	Toenail Se	Lower Se in PE; more severe PE (delivery <32 weeks’) with lower Se within the PE group; higher PE risk in the bottom tertile of Se (OR 4.4, 95% CI 1.6–14.9)
[103]	China 2001	251 (98 IGT, 46 GDM, 90 healthy, 17 healthy non-pregnant)	20 and 42 weeks	Serum Se	Lower Se at 33–42 weeks’ than at 20–33 weeks’ in all pregnant women; lower Se in IGT and GDM; lower Se in healthy pregnancies compared to healthy non-pregnant

CI, Confidence Interval; GDM, Gestational diabetes mellitus; GPx, Glutathione Peroxidase; GSH, Glutathione; GST, Glutathione S-transferase; Hct, Haematocrit; IDDP, Insulin-Dependent Diabetes mellitus in Pregnancy; IGT, Impaired glucose tolerance; IUGR, Intrauterine growth restriction; MDA, Malondialdehyde; μg/d, micrograms per day; OP, Oligohydramniotic Pregnancy; OR, Odds Ratio; PAP, Pregnancy with Abruption Placentae; PE, Preeclampsia; PIH, Pregnancy-induced hypertension; PROM, Premature rupture of membrane; PTB, Preterm birth; RBC, Red blood cell; RCT; Randomized controlled trial; Se, selenium; SOD, superoxide dismutase; TBARS, Thiobarbituric acid reactive substances.

**Table 3:** Iodine status and pregnancy complications.

Ref.	Country Year	Sample Size (Pregnant Women)	Time of Sampling	Sample Type and Assays	Outcome
[153]	UK 2018	3182 (3140 with child alive at 1st year, 42 pregnancy/infant loss)	Not reported	Urinary iodine-to-creatinine ratio(spot urine)	No association between iodine status and pregnancy complications or infant loss
[69]	China 2018	1569 euthyroid and primapara	1st trimester	Urinary iodine concentration (spot urine)	Mild iodine deficiency (urinary iodine 100–150 μg/L) was an independent risk factor for GDM (OR 1.669, 95% CI 1.114–2.501, *p* < 0.05). More than adequate and excessive iodine (urinary iodine ≥250 μg/L) was an independent risk factor for macrosomia (OR = 2.116, 95% CI 1.218–3.676, *p* < 0.05)
[154]	China 2018	2347	1st, 2nd, 3rd trimester	Urinary iodine concentration (spot urine)	Lower incidences of PE in pregnant women with UIC 150–249 μg/L compared to the reference group of UIC < 50 μg/L (OR = 0.12, 95% CI 0.01–0.87, *p* < 0.05)
[66]	Mexico 2017	57 (37 normotensive, 20 HPD)	3rd trimester	Urinary iodine concentration (spot urine), SOD, CAT, TBARS	Significant association between iodine deficiency and hypertensive disease of pregnancy (HPD); lower level of urinary iodine, SOD and CAT and higher level of TBARS in HPD compared to normotensive
[64]	Thailand 2016	390	Each trimester	Urinary iodine concentration (spot urine)	Higher PTB risk (OR 2.69, 95% CI 1.38–5.24, *p* = 0.004) and low birthweight (OR 2.66, 95% CI 1.40–5.05, *p* = 0.003) among urinary iodine < 150 μg/L
[155]	Argentina 2012	77	Not reported	Urinary iodine concentration (morning and evening urine samples, placental weight, placental index	Higher risk of lower placental weight in iodine deficiency (urinary iodine < 150 μg/L) (OR 3, 95% CI 1.06–8.5)
[156]	Turkey 2010	58 (40 severe PE, 18 healthy)	Not reported	Urinary iodine concentration (spot urine), thyroid hormone levels (T3, T4, TSH, fT3, fT4), blood magnesium	Positive correlation between urinary iodine and blood magnesium level in PE; higher T3 and fT3 levels in PE
[144]	Turkey 2009	40 (24 severe PE, 16 healthy)	Not reported for blood	Serum protein-bound iodine	Lower level of serum protein-bound iodine in maternal blood in PE; higher serum protein-bound iodine level in umbilical cord blood of infants in severe PE
[65]	Turkey 2007	35 (20 severe PE, 15 healthy)	Not reported for blood	Placental tissue iodine content, blood magnesium	Lower placental iodine in PE; positive correlation between placental iodine and blood magnesium level in PE
[145]	Senegal 2000	882 (462 pregnant, 420 non-pregnant)	Not reported	Urinary iodine concentration (spot urine), rate of miscarriage and stillbirth	Higher risk of miscarriage and stillbirth in iodine deficiency; highest rate in severe iodine deficiency (urinary iodine ≤ 20 μg/L) (OR 3.64, 95% CI 2.92–4.55)
[157]	China 1997	>60,000 Iodine supplementation to water	Not reported	Neonatal and infant mortality rate after iodine supplementation	Large reduction in both neonatal and infant mortality with iodine supplementation of water among all population in three severely iodine-deficient townships

CAT, Catalase; CI, Confidence Interval; fT3, Free triiodothyronine; fT4, Free thyroxine; GDM, Gestational diabetes mellitus; IUGR, Intra uterine growth restriction; μg/L, micrograms per litre; OR, Odds Ratio; PE, Preeclampsia; PIH, Pregnancy-induced hypertension; PTB, Preterm birth; SOD, superoxide dismutase; T3, triiodothyronine; T4, thyroxine; TBARS, Thiobarbituric acid reactive substances; TSH, thyroid-stimulating hormone; UIC, Urinary iodine concentration.
